# A biclustering algorithm based on a Bicluster Enumeration Tree: application to DNA microarray data

**DOI:** 10.1186/1756-0381-2-9

**Published:** 2009-12-16

**Authors:** Wassim Ayadi, Mourad Elloumi, Jin-Kao Hao

**Affiliations:** 1 UTIC, Higher School of Sciences and Technologies of Tunis, 1008 Tunis, Tunisia; 2 LERIA, Université d'Angers, 2 Boulevard Lavoisier, 49045 Angers, France

## Abstract

**Background:**

In a number of domains, like in DNA microarray data analysis, we need to cluster simultaneously rows (genes) and columns (conditions) of a data matrix to identify groups of rows coherent with groups of columns. This kind of clustering is called *biclustering*. Biclustering algorithms are extensively used in DNA microarray data analysis. More effective biclustering algorithms are highly desirable and needed.

**Methods:**

We introduce *BiMine*, a new enumeration algorithm for biclustering of DNA microarray data. The proposed algorithm is based on three original features. First, *BiMine *relies on a new evaluation function called *Average Spearman's rho *(ASR). Second, *BiMine *uses a new tree structure, called *Bicluster Enumeration Tree *(BET), to represent the different biclusters discovered during the enumeration process. Third, to avoid the combinatorial explosion of the search tree, *BiMine *introduces a parametric rule that allows the enumeration process to cut tree branches that cannot lead to good biclusters.

**Results:**

The performance of the proposed algorithm is assessed using both synthetic and real DNA microarray data. The experimental results show that *BiMine *competes well with several other biclustering methods. Moreover, we test the biological significance using a gene annotation web-tool to show that our proposed method is able to produce biologically relevant biclusters. The software is available upon request from the authors to academic users.

## Background

DNA microarray technology is a revolutionary method enabling the measurement of expression levels of at least thousands of genes in a single experiment under diverse experimental conditions. This technology has found numerous applications in research and applied areas like biology, drug discovery, toxicological study and diseases diagnosis.

DNA microarray data is typically represented by a matrix where each cell represents the gene expression level of a gene under a particular experimental condition. One important analysis task of microarray data concerns the simultaneous identification of groups of genes that show similar expression patterns across specific groups of experimental conditions (samples) [1]. Such an application can be addressed by a biclustering process whose aim is to discover coherent biclusters. That is, a bicluster is a subset of genes and conditions of the original expression matrix where the selected genes present a coherent behavior under all the experimental conditions contained in the bicluster.

More generally, biclustering has also applications in other domains such as text mining [2,3], target marketing [4,5], markets search [6], search in databases [7,8] and analyzing foreign exchange data [9].

Formally, let *I *= {1, 2, ..., *n*} denote the index set of *n *genes and *J *= {1, 2, ..., *m*} the index set of *m *conditions, a *data matrix M*(*I*, *J*) associated with *I *and *J *is a n*m matrix where the *i*^th ^row, *i *∈ *I*, represents the *i*^th ^gene or attribute and the *j*^th^, *j *∈ *J*, column represents the *j*^th ^condition or individual and *m*_*ij *_of the *i*^th ^row and the *j*^th ^column represents the value of the *j*^th ^condition for the *i*^th ^gene. A *bicluster *in a data matrix *M*(*I*, *J*) is a couple (*I*', *J*') such that *I*'⊆ *I *and *J*'⊆ *J*. The biclustering problem can be formulated as follows: Given a data matrix *M*, construct a bicluster *B*_*opt *_associated with *M *such that:(1)

where *f *is an *objective function *measuring the *quality*, i.e., degree of coherence, of a group of biclusters and *BC*(*M*) is the set of all the possible groups of biclusters associated with *M*.

Clearly, biclustering is a highly combinatorial problem with a search space of order of *O*(*2*^|*I*|+|*J*|^). In the general case, biclustering is known to be NP-hard [1]. Consequently, most of the algorithms used to discover biclusters are based on heuristics to explore partially the combinatorial search space. The existing algorithms for biclustering can roughly be classified into two large families: systematic search methods and stochastic search methods (also called metaheuristic methods). Representative examples of systematic search methods include, among others, greedy algorithms [1,10-14], divide and conquer algorithms [7,15] and enumeration algorithms [16-18]. On the other hand, among the metaheuristic methods, we can mention neighbourhood-based algorithms like simulated annealing [19], GRASP [20], evolutionary and hybrid algorithms [21-24]. A recent review of various biclustering algorithms for biological data analysis is provided in [25].

Since the biclustering problem is a NP-hard problem and no single existing algorithm is completely satisfactory for solving the problem. It is useful to seek more effective algorithms for better solutions. In this paper, we introduce a new enumeration algorithm for biclustering of DNA microarray data, called *BiMine*. Our algorithm is based on three original features. First, *BiMine *relies on a new evaluation function called *Average Spearman's rho *(ASR) which is used to guide effectively the exploration of the search space. Second, *BiMine *uses a new tree structure, called *Bicluster Enumeration Tree *(BET), to represent conveniently the different biclusters discovered during the enumeration process. Third, to avoid the combinatorial explosion of the search tree, *BiMine *introduces a parametric rule that allows the enumeration process to cut tree branches that cannot lead to good biclusters.

To assess the performance of the proposed *BiMine *algorithm, we show computational results obtained on both synthetic and real datasets and compare our results with those from four state-of-the-art biclustering algorithms. Moreover, to evaluate the biological relevance of our resulting biclusters, we carry out a practical validation with respect to a specific Gene Ontology (GO) annotation with the help of a popular web tool.

## Methods

### A New Evaluation Function of Biclustering

Like any search algorithm, *BiMine *needs an evaluation function to assess the quality of a candidate bicluster. One possibility is to use the so-called *Mean Squared Residue *(MSR) function [1]. Indeed, since its introduction, MSR has largely been used by biclustering algorithms, see for instance [11,13,20-22,26,27]. However, MSR is known to be deficient to assess correctly the quality of certain types of biclusters [14,28,29]. In a recent work, Teng and Chan [14] proposed another function for bicluster evaluation called *Average Correlation Value *(ACV). However, the performance of ACV is known to be sensitive to errors [13].

In this paper, we propose a new evaluation function called *Average Spearman's rho *(ASR) based on *Spearman's rank correlation*. Let  and  be two vectors, the *Spearman's rank correlation *[30] expresses the dependency between the vectors *X*_*i *_and *X*_*j *_(denoted by *ρ*_*ij*_) and is defined as follows:(2)

where  (resp. ) is the rank of  (resp. ).

Let (*I'*, *J'*) be a bicluster in data matrix *M*(*I*, *J*), the ASR evaluation function is then defined by:(3)

where:

*ρ*_*i*, *j *_(*i *≠ *j*) is the Spearman's rank correlation associated with the row indices *i *and *j *in the bicluster (*I'*, *J'*). *ρ*_*k*, *l *_(*k *≠ *l*) is the Spearman's rank correlation associated with the column indices *k *and *l *in the bicluster (*I'*, *J'*).

**Proposition 1: **Let (*I*', *J*') be a bicluster in a data matrix *M*(*I*, *J*). We have:

**Proof: **Let us first show that:

Indeed, we have  Spearman's rank correlations to calculate. According to [30], a Spearman's rank correlation belongs to [-1..1], we have then:

i.e.

It is easy to show in the same way that:

Hence:

i.e.:

With Spearman's rank correlation, a high (resp. low) value, *close *to 1 (resp. *close *to -1), indicates that the data is strongly (resp. weakly) correlated between two vectors [30]. As shown above, ASR also takes values from [-1..1]. A high (resp. low) ASR value, *close *to 1 (resp. *close *to -1), indicates that the genes/conditions of the bicluster are strongly (resp. weakly) correlated.

Furthermore, in the next subsection, we want to assess the quality of the proposed ASR evaluation function in comparison with two popular functions MSR and ACV.

### Studies of the ASR Evaluation Function

We compare the ASR evaluation function with *Mean Squared Residue *(MSR) [1]. As mentioned previously, MSR is probably the most popular evaluation function and largely used in the literature. As a second reference function, we use *Average Correlation Value *(ACV) which was proposed very recently in [14].

**Figure 1:**
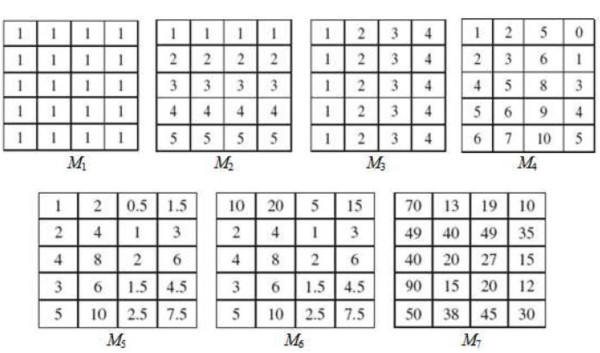
**Different typical Biclusters**. Data matrix *M*_1 _represents a constant bicluster, *M*_2 _represents a constant rows bicluster, *M*_3 _represents a constant column bicluster, *M*_4 _represents coherent values (additive model), *M*_5 _represents coherent values (multiplicative model), *M*_6 _represents coherent values (multiplicative model, where the first row of *M*_5 _is multiplied by 10) and *M*_7 _represents a coherent evolution.

For the comparison, we apply the evaluation functions (without using any algorithms), i.e., ASR, MSR and ACV, on seven matrices (biclusters) denoted by *M*_*1 *_to *M*_*7 *_(Figure 1). These matrices are employed in [14,25] and represent all typical biclusters. They are defined as follows. *M*_*1 *_is a constant bicluster, *M*_*2 *_has constant rows, *M*_*3 *_has constant columns, *M*_*4 *_is composed of coherent values (additive model), *M*_*5 *_represents coherent values (multiplicative model), *M*_*6 *_contains coherent values (multiplicative model, where the first row of *M*_*5 *_is multiplied by 10) and *M*_*7 *_represents a coherent evolution.

The values of ASR versus MSR and ACV are illustrated by Table 1 where the values of MSR and ACV were taken from [14].

**Table 1:** ASR versus MSR and ACV.

Biclusters	*M*_**1**_	*M*_**2**_	*M*_**3**_	*M*_**4**_	*M*_**5**_	*M*_**6**_	*M*_**7**_
Evaluation Functions							
MSR	0	0	0	0	0.62	2.425	131.87

ACV	1	1	1	1	1	1	0.84

ASR	1	1	1	1	1	1	0.99

Concerning MSR, a low (resp. high) value, *close *to 0 (resp. higher than a fixed threshold), indicates that the genes/conditions of the bicluster are strongly (resp. weakly) correlated.

Concerning ACV, a high (resp. low) value, *close *to 1 (resp. *close *to 0), indicates that the genes/conditions of the bicluster are strongly (resp. weakly) correlated.

According to Table 1, the ASR, ACV and MSR functions are perfect to assess the quality of biclusters *M*_1_, *M*_2_, *M*_3 _and *M*_4_. However, MSR is deficient on *M*_6 _and *M*_7_, confirming the claim that MSR may have trouble on certain types of biclusters [14,28,29]. On the other hand, ASR and ACV are perfect to assess the quality of biclusters *M*_5_and *M*_6 _but ASR is slightly better than ACV when applied on *M*_7_.

### BiMine Algorithm

We present now our biclustering algorithm called *BiMine *which uses ASR as its evaluation function and a new structure, called *Bicluster Enumeration Tree *(BET) to represent the different biclusters associated with a data matrix. We describe first the main procedure for building biclusters and then show an illustrative example to ease the understanding of the algorithm.

Let *M *be a data matrix, by using our algorithm, we operate in three steps: During the first step, we preprocess the data matrix *M*. During the second step, we construct a BET associated with *M*. Finally, during the last step, we identify the *best *biclusters.

#### Preprocessing

In the clustering area, preprocessing is often used to eliminate *insignificant *attributes (genes). For the biclustering, the preprocessing step aims to remove irrelevant expression values of the data matrix *M *that do not contribute in obtaining pertinent results. A value *m*_*ij *_of *M *is considered to be *insignificant *if we have:(4)

where *avg*_*i *_is the average over the non-missing values in the *i*^th ^row, *m*_*ij *_represents the intersection of row *i *with column *j *and *δ *is a fixed threshold. Equation 4 is applied for each value of *M*. See Tables 2 and 3 for an example.

**Table 2:** Data matrix *M'*.

	**C**_**1**_	**C**_**2**_	**C**_**3**_	**C**_**4**_	**C**_**5**_	**C**_**6**_
I_1_	10	20	5	15	40	18

I_2_	20	40	10	30	24	20

I_3_	23	12	8	15	29	50

I_4_	4	8	2	6	5	5

I_5_	15	25	8	12	29	50

**Table 3:** Data matrix *M *after preprocess.

	**C**_**1**_	**C**_**2**_	**C**_**3**_	**C**_**4**_	**C**_**5**_	**C**_**6**_
I_1_	10	20	5	15	40	-

I_2_	20	40	10	30	-	20

I_3_	-	12	8	15	29	50

I_4_	4	8	2	6	-	-

I_5_	15	-	8	12	29	50

By considering only non-missing values, we minimize the loss of information in the data matrix. This way of preprocessing missing values should be contrasted with other techniques. For instance, in [31], where the whole row is removed if the row contains at least one missing value or in [32], where the whole column is removed if it contains at least 5% of missing values. Furthermore, *BiMine *operates directly on the raw data matrix without resorting to a discretization of data, reducing thus the risk of loss of information.

#### Building Bicluster Enumeration Tree

After the preprocessing step, we construct a *Bicluster Enumeration Tree *(BET) that represents every possible bicluster that can be made from *M*. Compared to other data structure, BET permits to represent the maximum number of significant biclusters and the links that exist between these biclusters. Since the number of possible biclusters (nodes of BET) increases exponentially, *BiMine *employs parametric rules to help the enumeration process to close (or cut) a tree node. Intuitively, a node is cut down if the quality of the bicluster represented by this node is below a fixed threshold.

To describe formally our *BiMine *algorithm, let us define in the following the needed notations:

*n*_*i*_: *i*th node order containing biclusters.

*n*_*i*_.*g*_*i*_: genes of *n*_*i*_.

*n*_*i*_.*Cg*_*i*_: conditions of *n*_*i*_.

*bic*: bicluster.

*δ*: threshold used in Equation 4.

**Threshold**: quality threshold according to ASR.

**Figure 2:**
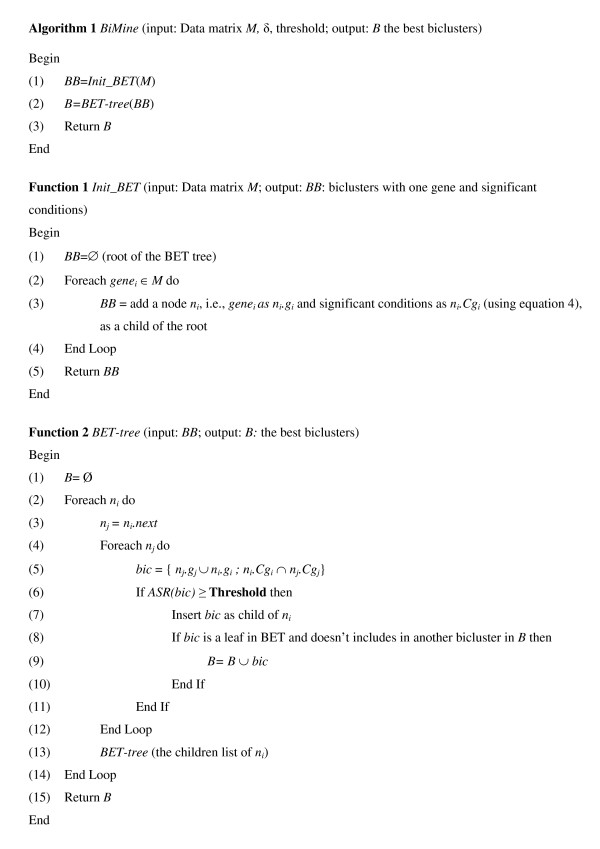
***BiMine *algorithm**.

The *BiMine *algorithm (Figure 2 (Algorithm 1)) uses a first function to built an initial tree (*Init_BET*) which is recursively extended by a second function (*BET-tree*). *Init_BET *(Figure 2 (Function 1)) generates thus the different biclusters from data matrix *M *with one gene and significant conditions after using Equation 4. The root of BET is the empty bicluster (Line 1). The nodes at level one are the possible biclusters with one gene (Line 2-4). Notice that each node *n*_*i *_is composed of two part *n*_*i*_.*g*_*i *_(genes) and *n*_*i*_.*Cg*_*i *_(significant conditions after the filter preprocessing with Equation 4). From these initial biclusters, new and larger biclusters are recursively built while pruning as soon as possible any bicluster if its ASR value doesn't reach a fixed Threshold. This is the role of the next function *BET-tree*.

*BET-tree *(Figure 2 (Function 2)) creates recursively the BET (Line 13) and generates the set of the best biclusters. The *i*^th ^child of a node is made up, on the one hand, of the *union *of the genes of the father node and the genes of the *i*^th ^uncle node, starting from the right side of the father. On the other hand, it is made up of the *intersection *of the conditions of the father and those of the *i*^th ^uncle starting from the right side of the father (Line 4-12). If the ASR value associated with the *i*^th ^child is smaller than or equal to the given *Threshold*, then this child will be ignored (Line 6-11).

Notice that this parametric pruning rule based on a quality threshold is fully justified in this context. Indeed, if the current bicluster is not good enough, then it is useless to keep it because expanding such a bicluster leads certainly to biclusters of worse quality. From this point of view, the pruning rule shares similar principles largely applied in optimization methods like Dynamic Programming. In addition, this pruning rule is essential in reducing the tree size and remains indispensable for handling large datasets.

Finally, the union of the leaves of the constructed BET that are not included in other leaves and have at least two genes represents a *good *group of biclusters (Line 8-9).

**Proposition 2**: Time complexity of *BiMine *is *O*(2^*n*^*mlog*(*m*)), where *n *is the number of rows and *m *is the number of columns of the data matrix.

**Proof: **Time complexity of the first step of *BiMine *is *O*(*nm*). Indeed, this step is achieved *via *a scanning of the whole data matrix *M *that is of size *nm*.

Time complexity of the second step of *BiMine *is *O*(2^*n*^*mlog*(*m*)). Actually, in the worst case, we have 2^*n *^nodes in the BET, representing the possible clusters of genes, each of which is associated with *m *conditions. On the other hand, since the conditions of the node are sorted, the construction of the intersection of two subsets of conditions of size *m *boils down to the search of *m *elements in a sorted array of size *m*. This can be done *via *a dichotomic search with a time complexity *O*(*mlog*(*m*)). Hence, the time complexity of the second step of *BiMine *is *O*(2^*n*^*mlog*(*m*)). Thus, The time complexity of *BiMine *is *O*(2^*n*^*mlog*(*m*)).

#### Illustrative Example

**Figure 3:**
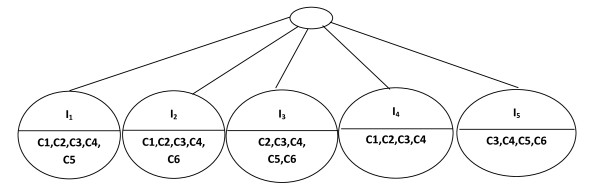
**First level of BET**.

Let *M' *a data matrix (Table 2). During the first step, we make a preprocessing of *M' *to obtain the data matrix *M *(Table 3). The character "-" represents a removed *insignificant *value. During the second step, we construct a BET that represents every possible bicluster that can be made from *M*. Let us set *δ *= 0.1 and threshold of ASR = 1. The first level of the BET is made up of the nodes that represent the possible biclusters with one gene. Each node represents a row of data matrix *M *(Figure 3).

The second level of the BET is made up of nodes that are the union of genes and the intersection of conditions in the first level.

**Figure 4:**
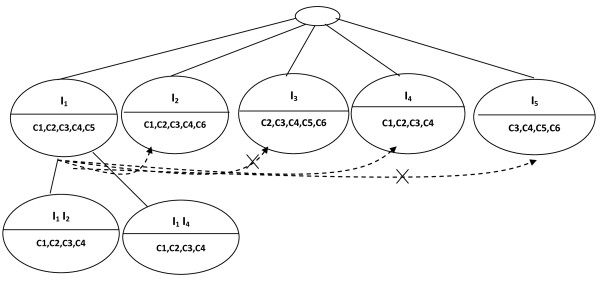
**Children construction of the first node of the second level of BET**.

In the Figure 4, we explain the construction of the children of node *I*_1_. Each dashed edges without cross represents a valid combination between two nodes (with ASR = 1). First, we perform the union of genes of node labeled *I*_*1 *_with those of *I*_*2 *_(first uncle), and the intersection of {c_1_, c_2_, c_3_, c_4_, c_5_} of *I*_*1 *_with those of {c_1_, c_2_, c_3_, c_4_, c_6_} of *I*_*2*_. The ASR of the obtained bicluster (*I*_*1*_, *I*_*2*_; c_1_, c_2_, c_3_, c_4_) is 1; hence we insert it as a first child of *I*_*1*_. After that, we process *I*_*1 *_with node labeled *I*_*3 *_(second uncle). We obtain the bicluster (*I*_*1*_, *I*_*3*_; c_2_, c_3_, c_4_, c_5_) with ASR lower than 1, hence, this child bicluster of *I*_*1 *_is discarded. We carry out the same process with node *I*_*4*_. We obtain the bicluster (*I*_*1*_, *I*_*4*_; c_1_, c_2_, c_3_, c_4_) with ASR equal to 1. We insert it as child of *I*_*1*_. Finally, with *I*_*5 *_we obtain the bicluster (*I*_*1*_, *I*_*5*_; c_1_, c_3_, c_4_, c_5_) with ASR lower than 1; hence we don't insert it.

**Figure 5:**
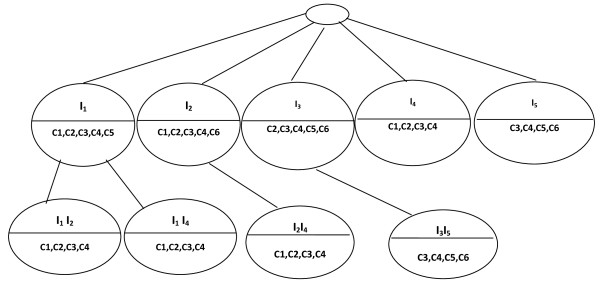
**Second level of BET**.

We repeat the same process for the node *I*_*2*_, *I*_*3*_, I_*4 *_and *I*_*5*_. This completes the second level of the BET (Figure 5).

**Figure 6:**
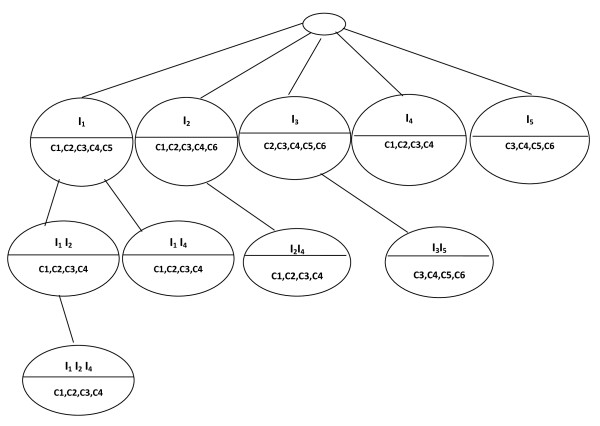
**Last level of BET**.

The third level of the BET is made up of nodes that are the union of genes and the intersection of conditions in the second level (Figure 6).

**Figure 7:**
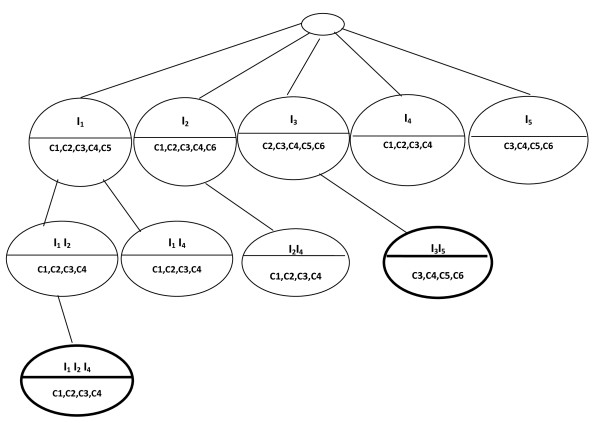
**Extracted biclusters are presented with bold line**.

At each level of the BET, we keep only nodes whose ASR is *equal *to 1. The union of the leaves of the constructed BET that are not included in other leaves is { (*I*_*1*_, *I*_*2*_, *I*_*4*_; c_1_, c_2_, c_3_, c_4_), (*I*_*3*_, *I*_*5; *_c_3_, c_4_, c_5_, c_6_) }. This constitutes the group of biclusters (Figure 7).

## Results

In this section, we assess the *BiMine *algorithm on both synthetic and real DNA microarray data. We have implemented our algorithm in Java programming language. We compare *BiMine *results with the results of four prominent biclustering algorithms used by the community, named as: CC [1], OPSM [10], ISA [33] and *Bimax *[15]. For these reference algorithms, we have used *Biclustering Analysis Toolbox *(BicAT) which is a recent software platform for clustering-based data analysis that integrates all these biclustering algorithms [34].

### Synthetic Data

#### Data Sets

According to [14,19,35], we generated randomly two types of synthetic datasets of size (I, J) = (200, 20). Different types of biclusters are embedded like constant columns, additive, multiplicative and coherent evolution biclusters. The first (resp. second) dataset contains biclusters without (resp. with) overlapping. To obtain statistically stable results, for each type of datasets, we generated 10 problem instances by randomly inserting the biclusters at different places in the data matrix.

#### Comparison Criteria

Following [35], we have used the following two ratios to evaluate our biclustering algorithm:(5)

with

*S*_*cb *_= Portion size of biclusters correctly extracted

*Tot*_*size *_= Total size of correct biclusters(6)

with

*S*_*ncb *_= Portion size of biclusters not correctly extracted

*Tot*_*size *_= Total size of corrected biclusters

The ratio *θ*_*Shared *_(resp. *θ*_*NotShared*_) expresses the percent of shared (resp. not shared) biclusters volume which corresponds (resp. not corresponds) with the real biclusters. In fact, when *θ*_*Shared *_(resp. *θ*_*NotShared*_) is equal to 100% the algorithm extracts the corrected (resp. not corrected) biclusters. A perfect solution have *θ*_*Shared *_= 100% and *θ*_*NotShared *_= 0%.

#### Protocol for Experiments

For our biclustering algorithm, we have fixed *δ *= 0.2 and threshold of ASR = 0.85. The parameter settings used for the four reference algorithms are the default values as used in [12]. We run all the algorithms and we select the 4 biclusters obtained by each algorithm which best fit the 4 real biclusters. We compute the *θ*_*Shared *_and the *θ*_*NotShared *_for each algorithm to show the averaged percentage of volume of the resulting biclusters which is shared and not shared with the real biclusters. The objective of this experiment is to determine which algorithm is able to extract all implanted biclusters.

Table 4 shows the best biclusters provided by each algorithm for the first dataset.

**Table 4:** *BiMine *results and comparison with other algorithms in synthetic data without overlapped biclusters.

Algorithms	***θ***_***Shared***_	***θ***_***NotShared***_
CC	18.21%	36.57%

OPSM	46.39%	74.42%

ISA	39.38%	5.31%

*Bimax*	58.18%	21.39%

*BiMine*	100%	33.03%

As we can see in Table 4, *BiMine *can extract 100% of implanted biclusters with an extra volume that represent 33,03% of implanted biclusters. In fact, to obtain a new bicluster, combining two biclusters provide an extra volume only on conditions but give exactly the correct number of genes. However, the best of the studied algorithms, i.e., *Bimax*, can extract only 58.18% of implanted biclusters with 21.39% of extra volume. CC uses the MSR function of the selected elements as the biclustering criterion. When the signal of the implanted biclusters is weak, the greedy nature of CC may delete some rows and columns of the implanted biclusters in the beginning of the algorithm and miss the deleted rows and columns in the output biclusters. ISA uses only up-regulated and down-regulated constant expression values in its biclustering algorithm. When coherent biclusters exist, ISA may miss some rows and columns of the implanted biclusters. OPSM seeks only up and down regulation expression values with coherent evolution. Its performance decreases when there exist scenarios constant biclusters. The discretization preprocessing used by *Bimax *cannot identify the elements in the coherent biclusters. Hence, the algorithm cannot find exactly the implanted biclusters.

Table 5 illustrates the best biclusters provided by each algorithm for the second dataset.

**Table 5:** *BiMine *results and comparison with other algorithms in synthetic data with overlapped biclusters.

Algorithms	***θ***_***Shared***_	***θ***_***NotShared***_
CC	9.21%	47.94%

OPSM	42.87%	49.31%

ISA	23.28%	23.97%

*Bimax*	34.07%	3.43%

*BiMine*	85.35%	41.78%

As we can see in Table 5, the results with *BiMine *present the highest coverage of the correct biclusters. In fact, *BiMine *can extract 85.35% of implanted biclusters with an extra volume that represent 41.78% of implanted biclusters. However, the best of the studied algorithms, i.e., OPSM, can extract only 42.87% of implanted biclusters with 49.31% of extra volume. To find overlapped biclusters in a given matrix, some algorithms, e.g., CC, need to mask the discovered biclusters with random values which is not necessary for *BiMine*. ISA and OPSM are sensitive to overlapping biclusters. They use the normalization step in the first preprocessing step of their algorithms. With overlapping biclusters, the expression value range after normalization becomes narrower. Table 5 shows that *BiMine *is marginally affected by the implanted overlap biclusters. We can conclude that *BiMine *can extract all implanted biclusters unlike other algorithms that can extract only certain types of biclusters.

### Real data

#### Data Sets

We applied our approach to the well-known yeast cell-cycle dataset. This dataset is publicly available from [36] and described in [37] and processed in [1]. It contains the expression profiles of more than 6000 yeast genes measured at 17 conditions over two complete cell cycles. In our experiments we use 2884 genes selected by [1].

#### Comparison Criteria

Two criteria are used. First, in order to evaluate the biological relevance of our proposed biclustering algorithm, we compute the *p*-values to indicate the quality of the extracted biclusters. Second, we identify the biological annotations for the extracted biclusters.

#### Protocol for Experiments

For our biclustering algorithm, we have fixed *δ *= 0.1 and threshold of ASR = 0.85. The parameter settings used for the different reference biclustering algorithms are the default settings as used in [12]. For the first experiment, we run all the algorithms and we compute the *p*-value for extracted biclusters. With *BiMine *(resp. *Bimax*), we have obtained more than 1800 (resp. 3700) biclusters. Since a biological analysis on 1800 (resp. 3700) biclusters was not feasible, only the 100 biggest biclusters with high ASR were selected for analysis like Christinat *et al*. [38]. Post-filtering was applied for all algorithms in order to eliminate insignificant biclusters like Cheng *et al*. [13]. With the others algorithms, we obtained 10 biclusters for CC, 45 biclusters for ISA and 14 biclusters for OPSM. For the second experiment, we use a well-known web-tool to search for the significant shared Gene Ontology terms of the groups of genes.

##### Biological relevance

In order to evaluate the biological relevance of our proposed biclustering algorithm, we compare it with the results of CC, ISA, *Bimax*, OPSM on yeast cell-cycle dataset. The idea is to determine whether the set of genes discovered by biclustering algorithms shows significant enrichment with respect to a specific Gene Ontology (GO) annotation. We use the web-tool *FuncAssociate *[39] to evaluate the discovered biclusters. *FuncAssociate *computes the adjusted significance scores for each bicluster. Indeed, the adjusted significance scores assess genes in each bicluster by computing adjusted *p*-values, which indicates how well they match with the different GO categories. Note that a smaller *p-*value, *close *to 0, is indicative of a better match [37]. Table 6 represents the different values of significant scores *p*-value for each algorithm over the percentage of total extracted biclusters. In fact with *BiMine*, 100% of tested biclusters have *p*-value = 5%. The same result is obtained with *p*-value = 1%. With *p*-value equals to 0.5% (resp. 0.1%), *BiMine *has 93% (resp. 82%) of biclusters. On the other hand, the best results (with the *p*-value is equals to 0.5% and 0.1% respectively) among the compared algorithms are obtained by *Bimax *with 89% (resp. 79%) of extracted biclusters. Finally, 51% of extracted biclusters with *BiMine *have *p*-value = 0.001% while those of *Bimax *have 64%. We note that *BiMine *performs well for all *p*-values compared to CC, ISA and OPSM. Also, *BiMine *performs well for four cases of *p*-value (*p*-value = 5%, *p*-value = 1%, *p*-value = 0.5% and *p*-value = 0.1%) over five compared to *Bimax*. Best results are obtained by *BiMine *and *Bimax*.

**Table 6:** Proportions of Biclusters significantly enriched by GO annotations.

p-value	5%	1%	0.5%	0.1%	0.001%
Algorithms					
*BiMine*	100	100	93	82	51

OPSM	100	100	86	36	22

*Bimax*	100	100	89	79	64

ISA	89	89	87	69	32

CC	80	70	60	20	10

Furthermore, in order to identify the biological annotations for the extracted biclusters we use *GOTermFinder *http://db.yeastgenome.org/cgi-bin/GO/goTermFinder which is a tool available in the *Saccharomyces Genome Database *(SGD). *GOTermFinder *is designed to search for the significant shared GO terms of the groups of genes and provides users with the means to identify the characteristics that the genes may have in common.

We present the significant shared GO terms (or parent of GO terms) used to describe the two selected set of genes (extracted by *BiMine*) with 11 genes × 11 conditions and 12 genes × 13 conditions in each bicluster with ASR equal to 0.8690 and 0.8873 respectively, for biological process, molecular function and cellular component. As [40], we report the most significant GO terms shared by these biclusters. For example, with the first bicluster (Table 7), the genes (*YDL003W, YDL164C, YDR097C, YDR440W, YKL113C, YLL002W, YLR183C, YNL102W*) are particularly involved in the process of cellular response to DNA damage stimulus, response to DNA damage stimulus, cellular response to stress, cellular response to stimulus, response to stress and response to stimulus.

**Table 7:** Most significant shared GO terms (process, function, component) for two biclusters on Yeast data.

Bicluster volume (genes × conditions)	Process Ontology	Function Ontology	Component Ontology
(12 × 13)	cellular response to DNA damage stimulus (66.7%, 1.87e-08) response to DNA damage stimulus (66.7%, 6.30e-08) cellular response to stress(66.7%, 2.12e-07) cellular response to stimulus(66,7%, 3.25e-07) DNA repair(50%, 2.58e-05) response to stress(66.7%, 2.98e-05)	chromatin binding (25%,0.00037)	microtubule organizing center part(16.7%, 0.00742)

(11 × 11)	cell cycle process (63.6%, 2.93e-05) cell cycle (63.6%, 6.85e-05)	GTPase activator activity (18.2%,0.00994)	microtubule cytoskeleton (45.5%, 6.33e-06) microtubule organizing center (36.4%,4.97e-05) spindle pole body (36.4%, 4.97e-05) spindle pole (36.4%, 6.77e-05)

The values within parentheses after each GO term in Table 7, such as (66.7%, 1.87e-08) in the first bicluster, indicate the cluster frequency and the statistical significance. The cluster frequency (66.7%) shows that out of 12 genes in the first bicluster 8 belong to this process, and the statistical significance is provided by a *p*-value of 1.87e-08 (highly significant).

**Figure 8:**
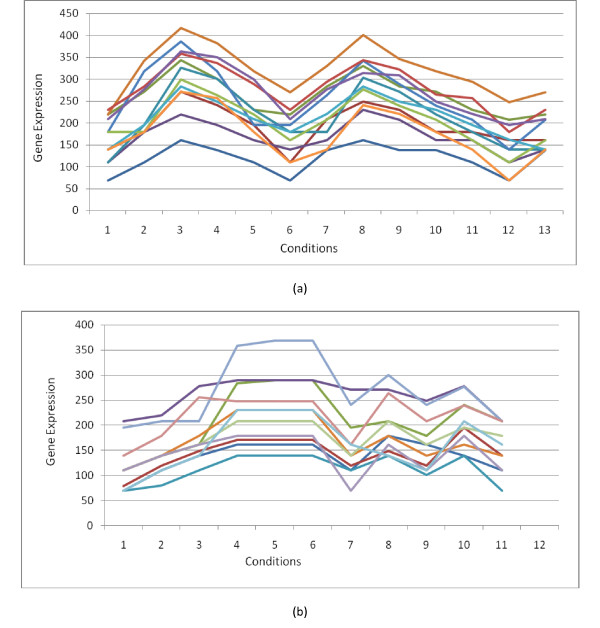
**Two Biclusters found by *BiMine *on Yeast dataset**. (a): Bicluster of size (12 × 13) with ASR = 0.8873. (b): Bicluster of size (11 × 11) with ASR = 0.8690.

According to [41-43], in microarray data analysis, genes are considered to be in the same cluster if their trajectory patterns of expression levels are similar across a set of conditions. Figure 8 shows the biclusters of Table 7 found by *BiMine *algorithm on the yeast dataset. From a visual inspection of the biclusters presented, we can notice that the genes present a similar behaviour under a subset of conditions. In Additional File 1, we show the best bicluster found by each compared algorithm using *GoTermFinder*. Also, we show their gene expression profiles drawn by BicAT. We notice that *BiMine *and *Bimax *have a high *p*-value. CC (resp. OPSM) cannot identify any component ontology (resp. function ontology) and ISA have *p*-value lower than *BiMine*.

All these experiments show that for this dataset, the proposed approach is able to detect biologically significant and functionally enriched biclusters with low *p*-value. Furthermore, *BiMine *gives a good degree of homogeneity.

## Discussion

*BiMine *algorithm has several interesting features. First, with *BiMine*, we avoid using a discretization of the data matrix. Indeed, classifying the gene expression values using intervals often leads to bad results [44]. Also, the discretization may limit the performance of an algorithm to discover a biological model because of noises which are inherent in most experiences of microarrays [31]. Thus, to discretize biological data we must have a good knowledge of these data to assign good values. However, this is not always possible.

Second, the *BiMine *algorithm can enumerate all possible cases of attributes while reducing the tree size. In fact, the parametric rule based on ASR threshold allows the enumeration process to prune tree branches that cannot lead to good biclusters.

Third, the *BiMine *algorithm provides naturally multiple biclusters of variable sizes. The number of the desired biclusters can be determined by tuning the ASR threshold. These multiple solutions of different sizes and different characteristics may be of interest for biological investigations.

Forth, the new ASR evaluation function can be applied by other biclustering algorithm in replacement of MSR or ACV. It can also be used as a complementary function to these previously ones.

Finally, in [45], it has been shown that Spearman's rank correlation is less sensitive to the presence of noise in the data. Since our evaluation function ASR is based on Spearman rank correlation, ASR would also be less sensitive to the presence of noise in the data.

## Conclusions

In this paper, we described *BiMine*, a new algorithm for biclustering of DNA microarray data. Compared with existing biclustering algorithms, *BiMine *distinguishes itself by a number of original features. First, *BiMine *operates directly on the raw data matrix without resorting to a discretization of data, reducing thus the risk of loss of information. Second, with *BiMine*, it is not necessary to fix a minimum or maximum number of genes or conditions, enabling the generation of diversified biclusters. Third, using a convenient tree structure for representing biclusters with a parametric and effective branch pruning rule, *BiMine *is able to explore effectively the search space. Notice that ASR can also be used by other biclustering algorithm as an alternative evaluation function.

The performance of the *BiMine *algorithm is tested and assessed on a set of synthetic data as well as a real microarray data (yeast cell-cycle). Computational experiments showed highly competitive results of *BiMine *in comparison with four other popular biclustering algorithms for both types of datasets. In addition, a biological validation of the selected genes within the biclusters for yeast cell-cycle has been provided based on a publicly available Gene Ontology (GO) annotation tool. Notice that although we presented *BiMine *with the context of DNA microarray data analysis, it should be clear that the algorithm can be applied or adapted to other biclustering problems.

Finally, let us mention that the proposed algorithm is computational time expensive; one of our ongoing works aims to find new heuristics to speed up the enumeration process. In particular, it would be possible to define other heuristic rules to improve the branch pruning in order to further reduce the size of the explored search tree.

## Competing interests

The authors declare that they have no competing interests.

## Authors' contributions

WA implemented the system, conducted the experimentations and wrote the draft manuscript. ME and JKH supervised the project and co-wrote the manuscript. All authors read and approved the final manuscript.

## Supplementary Material

Additional file 1 **The best bicluster obtained by each compared algorithm**. This file illustrates the best bicluster found by each compared algorithm using *GoTermFinder*. The gene expression profile of each best bicluster is drawn using BicAT. Click here for file
