# Supplementary material for: A biclustering algorithm based on a Bicluster Enumeration Tree: application to DNA microarray data
Source: BioData Min. 2009 Dec 16;2:9. doi: 10.1186/1756-0381-2-9 (PMC2804695; doi:10.1186/1756-0381-2-9)
Supplement: Additional file 1 — The best bicluster obtained by each compared algorithm. This file illustrates the best bicluster found by each compared algorithm using GoTermFinder. The gene expression profile of each best bicluster is drawn using BicAT. [file 1756-0381-2-9-S1.DOC]

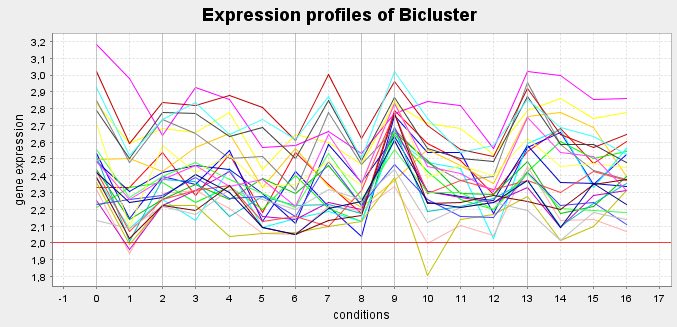


Figure 1: The bicluster found in the yeast dataset by *Bimax* (41×7)


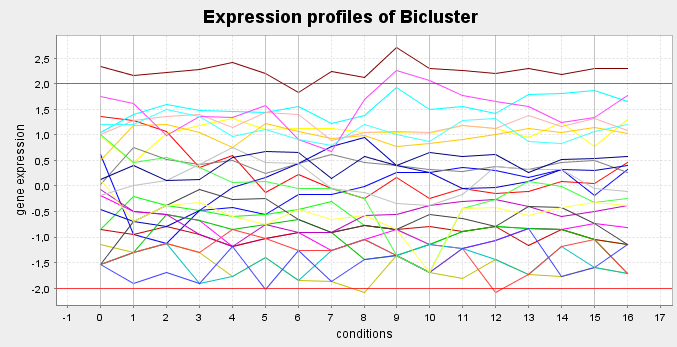


Figure 2: The bicluster found in the yeast dataset by CC (35×8)


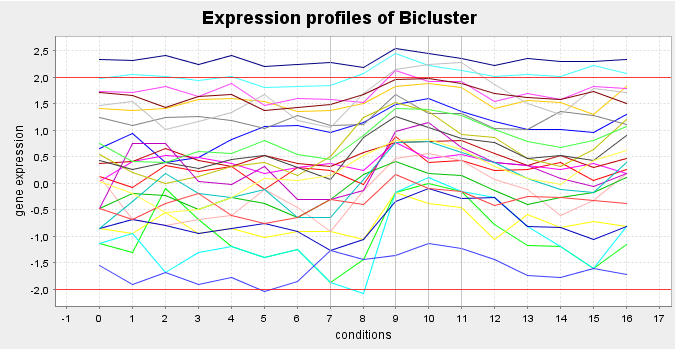


Figure 3: The bicluster found in the yeast dataset by *ISA* (28×4)


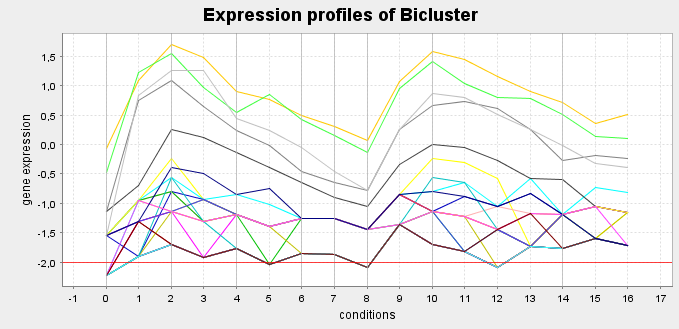
 Figure 4: The bicluster found in the yeast dataset by *OPSM* (25×8)

## Table 1 - Most significant shared GO terms (process, function, component) for the biclusters on Yeast data found by each compared algorithm

| Algorithms | Bicluster volume (genes ×  conditions) | Process  Ontology | Function  Ontology | Component  Ontology |
| --- | --- | --- | --- | --- |
| Bimax | 41×7 | [translation](http://www.yeastgenome.org/cgi-bin/GO/goTerm.pl?goid=6412) (73.2%, 2.71e-20)  [cellular biosynthetic process](http://www.yeastgenome.org/cgi-bin/GO/goTerm.pl?goid=44249) (85.4%, 1.30e-12) | [structural constituent of ribosome](http://www.yeastgenome.org/cgi-bin/GO/goTerm.pl?goid=3735)(68.3%, 2.20e-32)  [structural molecule activity](http://www.yeastgenome.org/cgi-bin/GO/goTerm.pl?goid=5198)(70.7%, 1.04e-28) | [cytosol](http://www.yeastgenome.org/cgi-bin/GO/goTerm.pl?goid=5829)(75.6%,8.73e-23)  [cytosolic large ribosomal subunit](http://www.yeastgenome.org/cgi-bin/GO/goTerm.pl?goid=22625)(43.9%,2.75e-22) |
| CC | 35×8 | [protein localization to chromosome](http://www.yeastgenome.org/cgi-bin/GO/goTerm.pl?goid=34502)(5.7%, 0.09723) | [3-hydroxyacyl-[acyl-carrier-protein] dehydratase activity](http://www.yeastgenome.org/cgi-bin/GO/goTerm.pl?goid=19171)(5.7%, 0.00049) | cellular component unknown |
| ISA | 28×4 | [telomere maintenance via recombination](http://www.yeastgenome.org/cgi-bin/GO/goTerm.pl?goid=722) (10.7%,0.00427)  [mitotic recombination](http://www.yeastgenome.org/cgi-bin/GO/goTerm.pl?goid=6312)(10.7%,0.07071) | [helicase activity](http://www.yeastgenome.org/cgi-bin/GO/goTerm.pl?goid=4386) (17.9%,0.00024)  [DNA helicase activity](http://www.yeastgenome.org/cgi-bin/GO/goTerm.pl?goid=3678)(10.7%, 0.00393) | [cellular bud](http://www.yeastgenome.org/cgi-bin/GO/goTerm.pl?goid=5933)(2.4%,0.01472) |
| OPSM | 25×8 | [cell cycle](http://www.yeastgenome.org/cgi-bin/GO/goTerm.pl?goid=7049) (7.3%,0.00065)  [regulation of cell cycle](http://www.yeastgenome.org/cgi-bin/GO/goTerm.pl?goid=51726) (24.0%,0.00218) | molecular function unknown | [condensed nuclear chromosome](http://www.yeastgenome.org/cgi-bin/GO/goTerm.pl?goid=794) (24.0%,1.74e-05)  [condensed chromosome](http://www.yeastgenome.org/cgi-bin/GO/goTerm.pl?goid=793) (24.0%,3.47e-05) |
